# Bis[bis­(2-methyl­phen­yl)phosphan­yl]methane

**DOI:** 10.1107/S1600536810054279

**Published:** 2011-01-08

**Authors:** Omar bin Shawkataly, Imthyaz Ahmed Khan, H. A. Hafiz Malik, Chin Sing Yeap, Hoong-Kun Fun

**Affiliations:** a Chemical Sciences Programme, School of Distance Education, Universiti Sains Malaysia, 11800 USM, Penang, Malaysia; b X-ray Crystallography Unit, School of Physics, Universiti Sains Malaysia, 11800 USM, Penang, Malaysia

## Abstract

In the title compound, C_29_H_30_P_2_, the dihedral angles between the two substituted benzene rings to the same P atom are 88.39 (7) and 83.88 (9)°. In the crystal, mol­ecules are arranged into columns and stacked down the *b* axis. Weak inter­molecular C—H⋯π inter­actions stabilize the crystal structure.

## Related literature

For related structures, see: Filby *et al.* (2006 ▶); Lumbreras *et al.* (2010 ▶). For the synthesis of bis­(di-*o*-tolyl­phosphino)methane, see: Filby *et al.* (2006 ▶). For the stability of the temperature controller used in the data collection, see: Cosier & Glazer (1986 ▶).
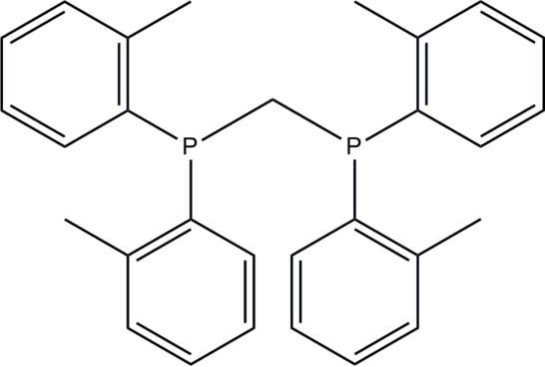


## Experimental

### 

#### Crystal data

C_29_H_30_P_2_
*M* *_r_* = 440.47
Monoclinic, 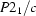

*a* = 8.2991 (5) Å
*b* = 7.4050 (5) Å
*c* = 40.782 (3) Å
β = 95.189 (1)°
*V* = 2496.0 (3) Å^3^
*Z* = 4
Mo *K*α radiation
μ = 0.19 mm^−1^
*T* = 100 K
0.43 × 0.42 × 0.10 mm

#### Data collection

Bruker APEXII DUO CCD area-detector diffractometer
Absorption correction: multi-scan (*SADABS*; Bruker, 2009 ▶) *T*_min_ = 0.924, *T*_max_ = 0.981
27134 measured reflections
10066 independent reflections
8035 reflections with *I* > 2σ(*I*)
*R*_int_ = 0.041

#### Refinement

*R*[*F* ^2^ > 2σ(*F* ^2^)] = 0.054
*wR*(*F* ^2^) = 0.137
*S* = 1.07
10066 reflections
284 parameters
H-atom parameters constrained
Δρ_max_ = 0.36 e Å^−3^
Δρ_min_ = −0.23 e Å^−3^

### 

Data collection: *APEX2* (Bruker, 2009 ▶); cell refinement: *SAINT* (Bruker, 2009 ▶); data reduction: *SAINT*; program(s) used to solve structure: *SHELXTL* (Sheldrick, 2008 ▶); program(s) used to refine structure: *SHELXTL*; molecular graphics: *SHELXTL*; software used to prepare material for publication: *SHELXTL* and *PLATON* (Spek, 2009 ▶).

## Supplementary Material

Crystal structure: contains datablocks global, I. DOI: 10.1107/S1600536810054279/ng5093sup1.cif

Structure factors: contains datablocks I. DOI: 10.1107/S1600536810054279/ng5093Isup2.hkl

Additional supplementary materials:  crystallographic information; 3D view; checkCIF report

## Figures and Tables

**Table 1:** Hydrogen-bond geometry (Å, °) *Cg*1 and *Cg*2 are the centroids of the C7–C12 and C20–C25 benzene rings, respectively.

*D*—H⋯*A*	*D*—H	H⋯*A*	*D*⋯*A*	*D*—H⋯*A*
C5—H5*A*⋯*Cg*1^i^	0.93	2.83	3.7325 (18)	164
C28—H28*A*⋯*Cg*2^ii^	0.96	2.76	3.6929 (18)	165

Symmetry codes: (i) 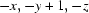; (ii) 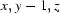.

**Fig. 1.**
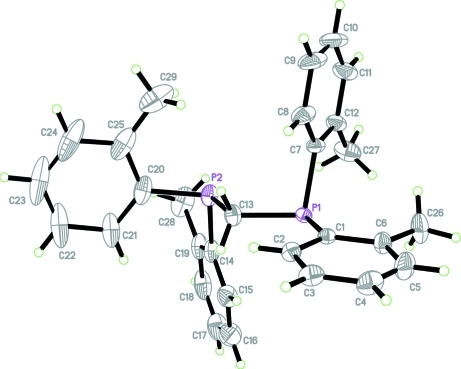
The molecular structure of the title compound with 50% probability ellipsoids for non-H atoms.

**Fig. 2.**
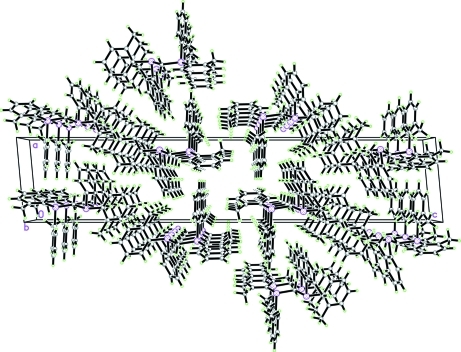
The crystal packing of the title compound, viewed down the b axis, showing the molecules stacked down the b axis.

## supplementary crystallographic information

### Comment

Diphosphines are an important class of ligands that finds widespread use in transition metal chemistry and catalysis. A subclass of these is small bite-angle diphosphines in which the two phosphorus centers are separated only by a single atom linker unit. The small bite-angle ligand bis(di-*o*-tolylphosphino)methane is used in the synthesis of palladium complexes (Filby *et al.*, 2006; Lumbreras  *et al.*, 2010).

The dihedral angles between the two substituted benzene rings (C1–C6/C7–C12 and C14–C19/C20–C25) to the same phosphine atom (P1 and P2) are 88.39 (7) and 83.88 (9)° respectively (Fig. 1). In the crystal packing, the molecules are arranged into columns and stacked down *b* axis. (Fig. 2). Weak intermolecular C—H···π interactions (Table 1) further stabilize the crystal structure.

### Experimental

All manipulations were performed under a dry oxygen-free nitrogen atmosphere using standard Schlenk techniques. All solvents were dried over sodium and distilled from sodium benzophenone ketyl under dry oxygen free nitrogen. Bis(di-*o*-tolylphosphino)methane was prepared by reported procedure (Filby *et al.*, 2006). Crystals suitable for X-ray diffraction were grown by slow solvent / solvent diffusion of CH_3_OH into CHCl_3_.

### Refinement

All hydrogen atoms were positioned geometrically and refined using a riding model with C—H = 0.93–0.97 Å and *U*_iso_(H) = 1.2 or 1.5 *U*_eq_(C). Rotating group model was applied for the methyl groups.

### Crystal data

**Table:**

C_29_H_30_P_2_	*F*(000) = 936
*M**_r_* = 440.47	*D*_x_ = 1.172 Mg m^−^^3^
Monoclinic, *P*2_1_/*c*	Mo *K*α radiation, λ = 0.71073 Å
Hall symbol: -P 2ybc	Cell parameters from 6549 reflections
*a* = 8.2991 (5) Å	θ = 4.6–33.8°
*b* = 7.4050 (5) Å	µ = 0.19 mm^−^^1^
*c* = 40.782 (3) Å	*T* = 100 K
β = 95.189 (1)°	Plate, colourless
*V* = 2496.0 (3) Å^3^	0.43 × 0.42 × 0.10 mm
*Z* = 4	

### Data collection

**Table:**

Bruker APEXII DUO CCD area-detector diffractometer	10066 independent reflections
Radiation source: fine-focus sealed tube	8035 reflections with *I* > 2σ(*I*)
graphite	*R*_int_ = 0.041
φ and ω scans	θ_max_ = 34.0°, θ_min_ = 4.3°
Absorption correction: multi-scan (*SADABS*; Bruker, 2009)	*h* = −13→12
*T*_min_ = 0.924, *T*_max_ = 0.981	*k* = −11→11
27134 measured reflections	*l* = −51→64

### Refinement

**Table:**

Refinement on *F*^2^	Primary atom site location: structure-invariant direct methods
Least-squares matrix: full	Secondary atom site location: difference Fourier map
*R*[*F*^2^ > 2σ(*F*^2^)] = 0.054	Hydrogen site location: inferred from neighbouring sites
*wR*(*F*^2^) = 0.137	H-atom parameters constrained
*S* = 1.07	*w* = 1/[σ^2^(*F*_o_^2^) + (0.0572*P*)^2^ + 0.7579*P*] where *P* = (*F*_o_^2^ + 2*F*_c_^2^)/3
10066 reflections	(Δ/σ)_max_ < 0.001
284 parameters	Δρ_max_ = 0.36 e Å^−^^3^
0 restraints	Δρ_min_ = −0.23 e Å^−^^3^

### Special details

**Table:**

Experimental. The crystal was placed in the cold stream of an Oxford Cryosystems Cobra open-flow nitrogen cryostat (Cosier & Glazer, 1986) operating at 100.0 (1) K.
Geometry. All e.s.d.'s (except the e.s.d. in the dihedral angle between two l.s. planes) are estimated using the full covariance matrix. The cell e.s.d.'s are taken into account individually in the estimation of e.s.d.'s in distances, angles and torsion angles; correlations between e.s.d.'s in cell parameters are only used when they are defined by crystal symmetry. An approximate (isotropic) treatment of cell e.s.d.'s is used for estimating e.s.d.'s involving l.s. planes.
Refinement. Refinement of *F*^2^ against ALL reflections. The weighted *R*-factor *wR* and goodness of fit *S* are based on *F*^2^, conventional *R*-factors *R* are based on *F*, with *F* set to zero for negative *F*^2^. The threshold expression of *F*^2^ > σ(*F*^2^) is used only for calculating *R*-factors(gt) *etc*. and is not relevant to the choice of reflections for refinement. *R*-factors based on *F*^2^ are statistically about twice as large as those based on *F*, and *R*- factors based on ALL data will be even larger.

### Fractional atomic coordinates and isotropic or equivalent isotropic displacement parameters (Å^2^)

**Table:**

	*x*	*y*	*z*	*U*_iso_*/*U*_eq_	
P1	0.18382 (3)	0.27891 (4)	0.086016 (7)	0.01724 (7)	
P2	0.16377 (4)	0.13495 (5)	0.154290 (8)	0.02634 (8)	
C1	0.23722 (13)	0.48860 (15)	0.06551 (3)	0.01841 (19)	
C2	0.29605 (15)	0.64220 (16)	0.08257 (3)	0.0230 (2)	
H2A	0.2991	0.6448	0.1054	0.028*	
C3	0.35022 (16)	0.79130 (18)	0.06591 (4)	0.0285 (3)	
H3A	0.3887	0.8928	0.0776	0.034*	
C4	0.34666 (19)	0.7880 (2)	0.03197 (4)	0.0342 (3)	
H4A	0.3834	0.8870	0.0207	0.041*	
C5	0.2885 (2)	0.6374 (2)	0.01471 (4)	0.0361 (3)	
H5A	0.2860	0.6367	−0.0081	0.043*	
C6	0.23309 (16)	0.48582 (19)	0.03092 (3)	0.0267 (2)	
C7	−0.03857 (13)	0.28696 (17)	0.08034 (3)	0.0217 (2)	
C8	−0.11985 (17)	0.4501 (2)	0.08482 (4)	0.0320 (3)	
H8A	−0.0605	0.5535	0.0907	0.038*	
C9	−0.28755 (19)	0.4594 (3)	0.08063 (5)	0.0438 (4)	
H9A	−0.3406	0.5678	0.0838	0.053*	
C10	−0.37401 (17)	0.3062 (3)	0.07174 (5)	0.0467 (5)	
H10A	−0.4864	0.3115	0.0689	0.056*	
C11	−0.29677 (17)	0.1445 (3)	0.06704 (4)	0.0399 (4)	
H11A	−0.3579	0.0428	0.0608	0.048*	
C12	−0.12682 (15)	0.13087 (19)	0.07148 (3)	0.0273 (2)	
C13	0.21569 (16)	0.33585 (17)	0.13027 (3)	0.0245 (2)	
H13A	0.1475	0.4370	0.1352	0.029*	
H13B	0.3277	0.3695	0.1360	0.029*	
C14	0.34812 (18)	−0.00360 (18)	0.15611 (3)	0.0282 (3)	
C15	0.48803 (19)	0.0451 (2)	0.14168 (4)	0.0352 (3)	
H15A	0.4921	0.1566	0.1313	0.042*	
C16	0.6214 (2)	−0.0687 (3)	0.14243 (5)	0.0464 (4)	
H16A	0.7136	−0.0331	0.1328	0.056*	
C17	0.6162 (3)	−0.2354 (3)	0.15766 (5)	0.0492 (5)	
H17A	0.7045	−0.3128	0.1581	0.059*	
C18	0.4799 (3)	−0.2860 (2)	0.17213 (4)	0.0451 (4)	
H18A	0.4779	−0.3978	0.1825	0.054*	
C19	0.3439 (2)	−0.17399 (19)	0.17171 (4)	0.0344 (3)	
C20	0.1778 (2)	0.23619 (19)	0.19566 (4)	0.0360 (3)	
C21	0.3219 (3)	0.2361 (2)	0.21606 (4)	0.0495 (5)	
H21A	0.4140	0.1830	0.2089	0.059*	
C22	0.3289 (4)	0.3153 (3)	0.24723 (5)	0.0738 (8)	
H22A	0.4254	0.3143	0.2607	0.089*	
C23	0.1940 (5)	0.3944 (3)	0.25798 (5)	0.0817 (10)	
H23A	0.1989	0.4464	0.2788	0.098*	
C24	0.0516 (4)	0.3970 (2)	0.23803 (6)	0.0698 (8)	
H24A	−0.0386	0.4528	0.2455	0.084*	
C25	0.0383 (3)	0.3179 (2)	0.20667 (5)	0.0476 (5)	
C26	0.1731 (2)	0.3240 (2)	0.01096 (4)	0.0415 (4)	
H26A	0.2340	0.2192	0.0184	0.062*	
H26B	0.1865	0.3450	−0.0119	0.062*	
H26C	0.0607	0.3047	0.0136	0.062*	
C27	−0.04883 (19)	−0.0484 (2)	0.06702 (5)	0.0401 (4)	
H27A	0.0148	−0.0813	0.0869	0.060*	
H27B	0.0197	−0.0410	0.0493	0.060*	
H27C	−0.1309	−0.1380	0.0619	0.060*	
C28	0.1983 (3)	−0.2342 (2)	0.18810 (5)	0.0504 (5)	
H28A	0.2161	−0.3540	0.1967	0.076*	
H28B	0.1051	−0.2339	0.1723	0.076*	
H28C	0.1806	−0.1532	0.2058	0.076*	
C29	−0.1195 (3)	0.3245 (3)	0.18557 (7)	0.0634 (6)	
H29A	−0.2006	0.3789	0.1977	0.095*	
H29B	−0.1521	0.2041	0.1793	0.095*	
H29C	−0.1067	0.3948	0.1662	0.095*	

### Atomic displacement parameters (Å^2^)

**Table:**

	*U*^11^	*U*^22^	*U*^33^	*U*^12^	*U*^13^	*U*^23^
P1	0.01554 (12)	0.01656 (13)	0.01936 (13)	0.00128 (9)	0.00012 (8)	0.00058 (9)
P2	0.03679 (18)	0.02088 (15)	0.02149 (15)	−0.00247 (12)	0.00350 (12)	0.00215 (11)
C1	0.0162 (4)	0.0182 (5)	0.0208 (5)	0.0014 (4)	0.0018 (3)	0.0014 (4)
C2	0.0241 (5)	0.0201 (5)	0.0250 (5)	0.0002 (4)	0.0038 (4)	−0.0015 (4)
C3	0.0270 (6)	0.0205 (5)	0.0387 (7)	−0.0026 (5)	0.0059 (5)	0.0002 (5)
C4	0.0359 (7)	0.0275 (6)	0.0403 (8)	−0.0046 (5)	0.0103 (6)	0.0096 (6)
C5	0.0486 (8)	0.0351 (7)	0.0249 (6)	−0.0052 (6)	0.0059 (6)	0.0076 (5)
C6	0.0310 (6)	0.0281 (6)	0.0209 (5)	−0.0033 (5)	0.0013 (4)	0.0020 (4)
C7	0.0164 (4)	0.0253 (5)	0.0237 (5)	0.0036 (4)	0.0039 (4)	0.0058 (4)
C8	0.0289 (6)	0.0316 (7)	0.0370 (7)	0.0123 (5)	0.0106 (5)	0.0081 (5)
C9	0.0287 (7)	0.0541 (10)	0.0511 (10)	0.0221 (7)	0.0167 (6)	0.0190 (8)
C10	0.0181 (6)	0.0732 (12)	0.0503 (10)	0.0098 (7)	0.0111 (6)	0.0275 (9)
C11	0.0209 (6)	0.0554 (10)	0.0430 (8)	−0.0091 (6)	0.0004 (5)	0.0179 (7)
C12	0.0188 (5)	0.0326 (6)	0.0299 (6)	−0.0043 (5)	0.0000 (4)	0.0079 (5)
C13	0.0326 (6)	0.0202 (5)	0.0204 (5)	−0.0005 (4)	0.0000 (4)	0.0021 (4)
C14	0.0431 (7)	0.0218 (5)	0.0191 (5)	0.0004 (5)	−0.0008 (5)	0.0007 (4)
C15	0.0389 (7)	0.0363 (7)	0.0293 (7)	0.0043 (6)	−0.0023 (5)	0.0083 (6)
C16	0.0424 (8)	0.0562 (11)	0.0395 (9)	0.0122 (8)	−0.0021 (7)	0.0064 (8)
C17	0.0603 (11)	0.0460 (10)	0.0387 (9)	0.0228 (9)	−0.0105 (8)	−0.0021 (7)
C18	0.0782 (13)	0.0264 (7)	0.0279 (7)	0.0123 (8)	−0.0111 (7)	−0.0009 (5)
C19	0.0600 (9)	0.0202 (5)	0.0222 (6)	0.0001 (6)	−0.0007 (6)	−0.0011 (4)
C20	0.0657 (10)	0.0208 (6)	0.0225 (6)	−0.0005 (6)	0.0102 (6)	0.0035 (5)
C21	0.0885 (14)	0.0334 (8)	0.0243 (7)	0.0016 (8)	−0.0067 (8)	−0.0027 (6)
C22	0.147 (3)	0.0442 (10)	0.0264 (8)	−0.0001 (13)	−0.0151 (11)	−0.0057 (8)
C23	0.183 (3)	0.0383 (10)	0.0264 (8)	0.0056 (14)	0.0223 (14)	−0.0045 (8)
C24	0.144 (2)	0.0280 (8)	0.0453 (11)	0.0098 (11)	0.0530 (14)	0.0054 (7)
C25	0.0845 (14)	0.0230 (6)	0.0401 (9)	0.0046 (8)	0.0322 (9)	0.0090 (6)
C26	0.0633 (10)	0.0380 (8)	0.0220 (6)	−0.0137 (7)	−0.0022 (6)	−0.0033 (6)
C27	0.0320 (7)	0.0270 (7)	0.0599 (11)	−0.0073 (6)	−0.0030 (6)	−0.0035 (7)
C28	0.0842 (14)	0.0236 (7)	0.0458 (10)	−0.0068 (8)	0.0188 (9)	0.0061 (6)
C29	0.0702 (14)	0.0477 (11)	0.0785 (16)	0.0154 (10)	0.0405 (12)	0.0136 (10)

### Geometric parameters (Å, °)

**Table:**

P1—C1	1.8368 (12)	C15—C16	1.389 (2)
P1—C7	1.8402 (11)	C15—H15A	0.9300
P1—C13	1.8491 (13)	C16—C17	1.384 (3)
P2—C14	1.8380 (15)	C16—H16A	0.9300
P2—C20	1.8404 (15)	C17—C18	1.375 (3)
P2—C13	1.8532 (13)	C17—H17A	0.9300
C1—C2	1.3981 (17)	C18—C19	1.399 (2)
C1—C6	1.4080 (17)	C18—H18A	0.9300
C2—C3	1.3922 (18)	C19—C28	1.500 (3)
C2—H2A	0.9300	C20—C21	1.394 (3)
C3—C4	1.382 (2)	C20—C25	1.415 (3)
C3—H3A	0.9300	C21—C22	1.396 (3)
C4—C5	1.382 (2)	C21—H21A	0.9300
C4—H4A	0.9300	C22—C23	1.371 (4)
C5—C6	1.4013 (19)	C22—H22A	0.9300
C5—H5A	0.9300	C23—C24	1.373 (4)
C6—C26	1.508 (2)	C23—H23A	0.9300
C7—C12	1.3982 (19)	C24—C25	1.402 (3)
C7—C8	1.4037 (18)	C24—H24A	0.9300
C8—C9	1.388 (2)	C25—C29	1.502 (4)
C8—H8A	0.9300	C26—H26A	0.9600
C9—C10	1.374 (3)	C26—H26B	0.9600
C9—H9A	0.9300	C26—H26C	0.9600
C10—C11	1.380 (3)	C27—H27A	0.9600
C10—H10A	0.9300	C27—H27B	0.9600
C11—C12	1.4093 (18)	C27—H27C	0.9600
C11—H11A	0.9300	C28—H28A	0.9600
C12—C27	1.495 (2)	C28—H28B	0.9600
C13—H13A	0.9700	C28—H28C	0.9600
C13—H13B	0.9700	C29—H29A	0.9600
C14—C15	1.396 (2)	C29—H29B	0.9600
C14—C19	1.4150 (19)	C29—H29C	0.9600
			
C1—P1—C7	101.29 (5)	C17—C16—C15	119.57 (18)
C1—P1—C13	103.45 (5)	C17—C16—H16A	120.2
C7—P1—C13	99.71 (6)	C15—C16—H16A	120.2
C14—P2—C20	101.95 (7)	C18—C17—C16	119.76 (17)
C14—P2—C13	103.71 (6)	C18—C17—H17A	120.1
C20—P2—C13	99.16 (6)	C16—C17—H17A	120.1
C2—C1—C6	119.09 (11)	C17—C18—C19	121.80 (16)
C2—C1—P1	123.29 (9)	C17—C18—H18A	119.1
C6—C1—P1	117.30 (9)	C19—C18—H18A	119.1
C3—C2—C1	121.06 (12)	C18—C19—C14	118.78 (16)
C3—C2—H2A	119.5	C18—C19—C28	119.95 (15)
C1—C2—H2A	119.5	C14—C19—C28	121.27 (15)
C4—C3—C2	119.74 (13)	C21—C20—C25	119.57 (16)
C4—C3—H3A	120.1	C21—C20—P2	121.78 (14)
C2—C3—H3A	120.1	C25—C20—P2	118.65 (15)
C5—C4—C3	119.95 (13)	C20—C21—C22	120.4 (2)
C5—C4—H4A	120.0	C20—C21—H21A	119.8
C3—C4—H4A	120.0	C22—C21—H21A	119.8
C4—C5—C6	121.37 (14)	C23—C22—C21	120.2 (3)
C4—C5—H5A	119.3	C23—C22—H22A	119.9
C6—C5—H5A	119.3	C21—C22—H22A	119.9
C5—C6—C1	118.79 (12)	C22—C23—C24	120.04 (19)
C5—C6—C26	119.24 (13)	C22—C23—H23A	120.0
C1—C6—C26	121.97 (12)	C24—C23—H23A	120.0
C12—C7—C8	119.90 (12)	C23—C24—C25	121.8 (2)
C12—C7—P1	120.11 (9)	C23—C24—H24A	119.1
C8—C7—P1	119.99 (10)	C25—C24—H24A	119.1
C9—C8—C7	120.98 (16)	C24—C25—C20	118.0 (2)
C9—C8—H8A	119.5	C24—C25—C29	120.1 (2)
C7—C8—H8A	119.5	C20—C25—C29	121.89 (17)
C10—C9—C8	119.04 (15)	C6—C26—H26A	109.5
C10—C9—H9A	120.5	C6—C26—H26B	109.5
C8—C9—H9A	120.5	H26A—C26—H26B	109.5
C9—C10—C11	121.03 (13)	C6—C26—H26C	109.5
C9—C10—H10A	119.5	H26A—C26—H26C	109.5
C11—C10—H10A	119.5	H26B—C26—H26C	109.5
C10—C11—C12	121.08 (16)	C12—C27—H27A	109.5
C10—C11—H11A	119.5	C12—C27—H27B	109.5
C12—C11—H11A	119.5	H27A—C27—H27B	109.5
C7—C12—C11	117.96 (14)	C12—C27—H27C	109.5
C7—C12—C27	122.89 (11)	H27A—C27—H27C	109.5
C11—C12—C27	119.14 (14)	H27B—C27—H27C	109.5
P1—C13—P2	108.26 (7)	C19—C28—H28A	109.5
P1—C13—H13A	110.0	C19—C28—H28B	109.5
P2—C13—H13A	110.0	H28A—C28—H28B	109.5
P1—C13—H13B	110.0	C19—C28—H28C	109.5
P2—C13—H13B	110.0	H28A—C28—H28C	109.5
H13A—C13—H13B	108.4	H28B—C28—H28C	109.5
C15—C14—C19	118.38 (14)	C25—C29—H29A	109.5
C15—C14—P2	124.06 (11)	C25—C29—H29B	109.5
C19—C14—P2	117.48 (12)	H29A—C29—H29B	109.5
C16—C15—C14	121.71 (15)	C25—C29—H29C	109.5
C16—C15—H15A	119.1	H29A—C29—H29C	109.5
C14—C15—H15A	119.1	H29B—C29—H29C	109.5
			
C7—P1—C1—C2	106.29 (10)	C14—P2—C13—P1	82.71 (7)
C13—P1—C1—C2	3.31 (11)	C20—P2—C13—P1	−172.51 (8)
C7—P1—C1—C6	−80.18 (10)	C20—P2—C14—C15	−102.79 (13)
C13—P1—C1—C6	176.84 (9)	C13—P2—C14—C15	−0.14 (14)
C6—C1—C2—C3	−0.01 (18)	C20—P2—C14—C19	80.55 (12)
P1—C1—C2—C3	173.42 (10)	C13—P2—C14—C19	−176.80 (10)
C1—C2—C3—C4	−0.3 (2)	C19—C14—C15—C16	−0.2 (2)
C2—C3—C4—C5	0.4 (2)	P2—C14—C15—C16	−176.83 (13)
C3—C4—C5—C6	−0.3 (2)	C14—C15—C16—C17	0.4 (3)
C4—C5—C6—C1	0.1 (2)	C15—C16—C17—C18	−0.6 (3)
C4—C5—C6—C26	−179.09 (16)	C16—C17—C18—C19	0.6 (3)
C2—C1—C6—C5	0.10 (18)	C17—C18—C19—C14	−0.4 (2)
P1—C1—C6—C5	−173.72 (11)	C17—C18—C19—C28	−179.46 (17)
C2—C1—C6—C26	179.24 (14)	C15—C14—C19—C18	0.2 (2)
P1—C1—C6—C26	5.43 (18)	P2—C14—C19—C18	177.07 (11)
C1—P1—C7—C12	137.48 (10)	C15—C14—C19—C28	179.22 (15)
C13—P1—C7—C12	−116.57 (11)	P2—C14—C19—C28	−3.92 (19)
C1—P1—C7—C8	−42.34 (12)	C14—P2—C20—C21	15.95 (15)
C13—P1—C7—C8	63.60 (11)	C13—P2—C20—C21	−90.27 (14)
C12—C7—C8—C9	0.1 (2)	C14—P2—C20—C25	−164.84 (11)
P1—C7—C8—C9	179.92 (12)	C13—P2—C20—C25	88.93 (12)
C7—C8—C9—C10	−0.5 (2)	C25—C20—C21—C22	0.3 (3)
C8—C9—C10—C11	0.1 (3)	P2—C20—C21—C22	179.50 (15)
C9—C10—C11—C12	0.6 (3)	C20—C21—C22—C23	−0.2 (3)
C8—C7—C12—C11	0.59 (19)	C21—C22—C23—C24	−0.4 (4)
P1—C7—C12—C11	−179.24 (11)	C22—C23—C24—C25	0.9 (3)
C8—C7—C12—C27	−178.62 (14)	C23—C24—C25—C20	−0.9 (3)
P1—C7—C12—C27	1.55 (19)	C23—C24—C25—C29	−179.73 (19)
C10—C11—C12—C7	−0.9 (2)	C21—C20—C25—C24	0.2 (2)
C10—C11—C12—C27	178.31 (16)	P2—C20—C25—C24	−178.99 (12)
C1—P1—C13—P2	179.41 (6)	C21—C20—C25—C29	179.07 (16)
C7—P1—C13—P2	75.22 (7)	P2—C20—C25—C29	−0.1 (2)

### Hydrogen-bond geometry (Å, °)

**Table:**

Cg1 and Cg2 are the centroids of the C7–C12 and C20–C25 benzene rings, respectively.

**Table:**

*D*—H···*A*	*D*—H	H···*A*	*D*···*A*	*D*—H···*A*
C5—H5A···Cg1^i^	0.93	2.83	3.7325 (18)	164
C28—H28A···Cg2^ii^	0.96	2.76	3.6929 (18)	165

Symmetry codes: (i) −*x*, −*y*+1, −*z*; (ii) *x*, *y*−1, *z*.
